# Current situation in Europe – different perspectives

**DOI:** 10.1192/j.eurpsy.2025.198

**Published:** 2025-08-26

**Authors:** M. Schouler-Ocak, Task Force on Migration and Mental Health

**Affiliations:** Psychiatry and Psychotherapy, Charité - Universitätsmedizin Berlin, Berlin, Germany

## Abstract

**Abstract:**

Germany is one of the countries that has taken in a large number of refugees. Around 2.25 million now have recognised protection status in Germany. In addition, Germany has taken in over 1,215,048 refugees from Ukraine. All of these people are very vulnerable refugees who are exposed to many risk and stress factors before, during and after their migration. As a result, they have a high prevalence of mental disorders such as post-traumatic stress disorder (PTSD), depression, anxiety, substance use and persistent grief disorder. At the same time, refugees face numerous barriers to accessing medical care, such as language and cultural barriers, administrative barriers, structural, institutional and interpersonal discrimination and racism. There is also unequal treatment in Germany between refugees from Ukraine and other regions of the world. In addition, healthcare provision has recently been tightened due to changes in the Asylum Seekers’ Benefits Act. This presentation will focus on the sitaution in Germany and discuss üossible solutions.

**Disclosure of Interest:**

None Declared

